# Hyperprogression in PDL1 Expressive, Recurrent Gastroesophageal-junction Adenocarcinoma After Pembrolizumab

**DOI:** 10.7759/cureus.4862

**Published:** 2019-06-07

**Authors:** Shashank Sama, Matthew J Hadfield, Nerea Lopetegui Lia, James Vredenburgh

**Affiliations:** 1 Internal Medicine, University of Connecticut Health, Farmington, USA; 2 Hematology and Oncology, Saint Francis Hospital and Medical Center, Hartford, USA

**Keywords:** hyperprogression, pembrolizumab, immune check point inhibitors, gastro-esophageal junction cancer

## Abstract

Hyperprogression is a pattern of accelerated tumor growth noted uncommonly after the use of immune checkpoint inhibitors in some patients. We present a 56-year-old female with gastroesophageal junction (GEJ) adenocarcinoma who was initially treated with neoadjuvant radiation and chemotherapy with carboplatin and paclitaxel, followed by esophagogastrectomy and postoperative FOLFOX chemotherapy. After a stable two-year course, she was noted to have recurrence at the GEJ which was biopsy confirmed. She was started on pembrolizumab, after which she developed several new metastases noted on the PET/CT. Lesions were noted in iliac bones, spine, retroperitoneal lymph nodes, hilar nodes, mediastinum, and lungs. Postdiscontinuation of the pembrolizumab, she received six cycles of paclitaxel with ramucirumab and showed remarkable improvement on the next imaging scan with resolution of osseous lesions, lung nodules and significant improvement in hilar, mediastinal, and retroperitoneal lymph nodes. We hope that this case report sheds further light on this uncommon complication of immune checkpoint inhibitors.

## Introduction

Immune checkpoint inhibitors have revolutionized cancer therapy in recent times. The increasing use of these novel agents for the treatment of neoplastic disease has brought to light new patterns of tumor response which were not previously seen with conventional chemotherapy. One of these uncommon phenomena is the pattern of hyperprogression. Hyperprogression is an atypical reaction of exaggerated tumor growth following immunotherapy. Even though hyperprogression is increasingly reported among other cancers, it is exceedingly rare among gastroesophageal junction (GEJ) tumors. The previously reported case in a GEJ tumor was in the setting of nivolumab use [1]. To our knowledge there has been no previously reported hyperprogression in GEJ tumors after pembrolizumab use and we present the first known occurrence of this in the case below.

## Case presentation

A 56-year-old female presented initially with complaints of intermittent dysphagia and a 15 lb. weight loss. The patient’s family history was significant for lung cancer in her mother while past medical history comprised primarily of hypertension, hyperlipidemia, and hypothyroidism. In addition, she also reported a 15-pack year smoking history and rare alcohol use. Her symptoms prompted an evaluation with an upper endoscopy that ultimately revealed an ulcerating mass in the distal esophagus. Biopsy of the lesion showed an adenocarcinoma, signet ring cell type which was poorly differentiated. The tumor was Her-2/neu negative by immunohistochemical staining and FISH. PET scan done at the time of initial evaluation showed intense metabolic uptake with an standardized uptake value (SUV) of 5.3 in the distal esophagus and GEJ with the CT scan showing a mass measuring 3.3 cm x 1.6 cm x 1.5 cm at the same site. There was no evidence of local or distant spread of tumor on this initial imaging and she was considered to be stage IIB, T3N0M0. 

Treatment was initiated with neoadjuvant chemoradiation with a total of seven weeks of full dose radiation therapy and weekly radio sensitizing chemotherapy with carboplatin and paclitaxel. An esophagogastrectomy done shortly after unfortunately showed extensive residual poorly differential adenocarcinoma with three out of 14 nodes being positive and invasion of the tumor into the adventitia which resulted in her being pathologically restaged as III B, pT3 pN2. Given the minimal pathologic response to the preoperative chemoradiation, it was decided to start her on adjuvant FOLFOX. She completed 12 cycles of FOLFOX, and repeat CT imaging after showed no recurrence or progression.

**Figure 1:**
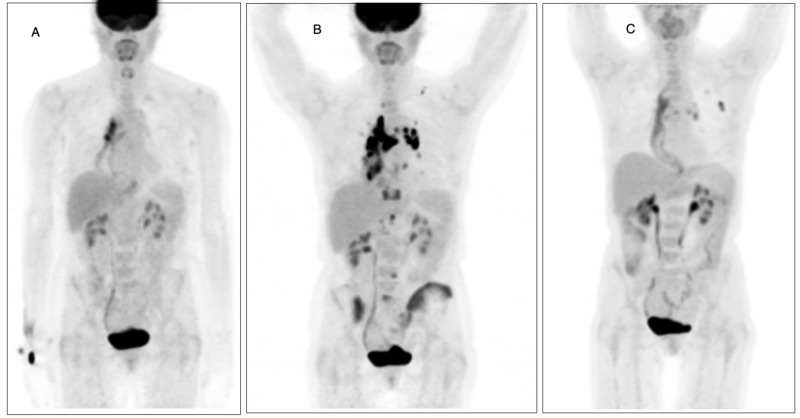
PET imaging in the patient before pembrolizumab, after starting pembrolizumab, and after discontinuation of pembrolizumab. A : PET imaging prior to initiation of pembrolizumab B: PET imaging after pembrolizumab therapy C: PET imaging after discontinuation of pembrolizumab and completion of six cycles of paclitaxel and ramucirumab

For two years following the completion of adjuvant chemotherapy, she had an unremarkable course with periodic CT imaging findings consistently negative for recurrent or metastatic disease. At the two-year mark, however, she began to develop symptoms of dysphagia for which she underwent esophagography showing interval narrowing of intrathoracic stomach. Endoscopy done at this time to further evaluate showed ulcerated and friable mucosa at the gastroesophageal anastomosis. Biopsy of the ulcerated lesion showed anastomotic recurrence of previously diagnosed poorly differentiated adenocarcinoma which was also positive for expression for PDL1. PET/CT imaging at this point showed nonspecific moderately intense metabolic activity within the region of anastomosis with no distant abnormal foci identified (Figure 1A). After discussing the potential for immunotherapy, she consented and was started on pembrolizumab. The patient tolerated the first cycle of pembrolizumab without any notable toxicities. However, after the second cycle she developed intermittent cough, dyspnea, and wheezing. Results of the chest X-ray and pulmonary function tests (PFTs) performed to further evaluate were unremarkable and her symptoms were attributed to be secondary to the gastric pull through surgery. Of particular note, on the day prior to her third cycle of pembrolizumab she developed cellulitis close to the chest port site and was prescribed Keflex for 10 days. She then received the third dose of pembrolizumab without interruption. On the 11th day post the third pembrolizumab dose she noticed a rash over the neck, trunk, and bilateral lower extremities which comprised pink, flat, and nonpruritic lesions. This was classified as a grade three maculopapular rash likely secondary to pembrolizumab or less likely the antibiotic. The rash resolved with steroids and a CT scan of the chest done at this point showed no evidence of pneumonitis and no focal airspace disease. She then went on to have an unremarkable cycle four of pembrolizumab with a planned staging PET/CT afterward. The PET scan after cycle four showed shocking evidence of extensive metastatic disease with intense focal uptake noted in several regions including multiple bones including iliac bones, four spinal lesions, upper retroperitoneal lymph nodes, several small lung metastases as well as extensive nodal involvement in the hilar regions and the mediastinum (Figure 1B). Increased uptake was also noted in the region of the surgical anastomosis. These findings of remarkable progression on immunotherapy were consistent with the pattern of hyperprogression after immunotherapy as described in literature. After discontinuation of pembrolizumab, the patient was started on ramucirumab and weekly paclitaxel to arrest any further progression. Ramucirumab and paclitaxel were chosen after reviewing the evidence from the Rainbow trial and case reports of response following immunotherapy [2]. After two cycles of this therapy the patient improved clinically and repeat CT imaging showed dramatic improvement from before with improved adenopathy in the chest and abdomen with no interval progression of metastatic disease. Given new bone metastases, bone strengthening therapy with Xgeva was also started. Apart from fingernail thickening she tolerated the therapy well, with re-staging PET scan after six cycles showing significant improvement of metastatic disease with resolution of fluorodeoxyglucose (FDG) uptake in osseous lesions and lung nodules and significant improvement in mediastinal and retroperitoneal FDG avid lymph nodes (Figure 1C). There was also improvement in FDG uptake around the gastroesophageal anastomosis. On completion of six cycles of paclitaxel, she was switched to maintenance ramucirumab with continued good response on the maintenance regimen.

## Discussion

Immune checkpoint inhibitors (ICIs) have changed the landscape of antineoplastic drugs, in large part by standing in contrast to traditional chemotherapy drugs due to their novel mechanism of action. These class of drugs act by blocking the interaction of cell-surface ligand and receptors, such as the PD1 and the CTLA 4 which are located on the cells of the immune system. The interaction of cell surface receptors and ligands is a major part of the immune checkpoint pathway, which has been previously known to play a role in the maintenance of self-tolerance and modulation of the immune response. The ICIs by blocking this interaction cause the opposite effect to the above immunomodulation, which is the amplification of the body’s immune mediated antitumor response [3]. Taking advantage of this unique approach of antitumor therapy, the ICIs have been deployed in the treatment of a variety of cancers such as non-small cell lung carcinomas, melanomas, Hodgkin’s lymphomas, and renal cell carcinomas [4-7]. They are now also increasingly being studied in other tumors including gastrointestinal tumors as well. Esophageal cancers with an estimated incidence of over 570,000 cases a year worldwide represent around 3.2% of all newly diagnosed cancers [8]. In the USA, around 75% of these esophageal cancers are primarily accounted by adenocarcinomas arising at the GEJ and the distal esophagus [9]. While surgical options along with perioperative chemotherapy have been the mainstay for treatment, ICIs are recently being evaluated in refractory disease. The ATTRACTION 2 phase III trial studied nivolumab versus placebo in Asian patients, while the phase II study KEYNOTE- 059 investigated the use of pembrolizumab [10-11]. Due to the promising results in the KEYNOTE -059 study pembrolizumab has since been FDA approved for patients with advanced gastric/GEJ adenocarcinoma whose tumors express PD L1 and have received greater than or equal to two chemotherapy regimens. In addition to this, nivolumab is also being studied in trial NCT02743494 for therapy in these tumors.

While the novel mode of action of the ICIs obviously differentiates the side effect profile of antineoplastic therapy from the classical chemotherapy drugs, these drugs also seem to differentiate themselves from known chemotherapy drugs by bringing to light new patterns of anti-tumor drug response not previously described in classical drugs. One of these unusual tumor response patterns is the phenomenon of hyperprogression. Hyperprogression is the seemingly paradoxical accelerated tumor growth observed after initiating therapy with ICI drugs. Hyperprogression has been variously defined under slightly different criteria. While Champiat et al. have incorporated a greater than or equal to two-fold increase in tumor growth rate post drug therapy as a part of their definition, Kato et al. have in addition included time to treatment failure of less than two months and a greater than 50% increase in tumor burden post therapy, as a part of their definition [12-13]. Hyperprogression has so far been increasing described in a wide variety of cancers including lung cancers, head and neck cancers urothelial carcinoma, and melanomas [14-16]. While hyperprogression has been previously documented in GEJ tumors after nivolumab, to our knowledge there has not been reportage of HP in GEJ tumors after usage of pembrolizumab as we have described in our case [1].

The incidence of hyperprogression in these reported data has ranged from 4% to 29% [17]. If there is indeed a differentiating factor among these subsets of patients which makes them prone for hyperprogression, it has yet to be conclusively determined. The exact mechanism underlying the phenomenon of hyperprogression also remains unclear at present, however, several theories have been proposed. Champiat et al. noted higher incidence in older patients especially in ages greater than 65, but our patient being younger would not fit under this profile [12]. Kato and colleagues meanwhile, in their paper suggested MDM2/MDM4 amplification and EGFR aberrations as a possible predisposing factor to hyperprogression [13]. Given the poor clinical status of our presented patient, we were unable to biopsy the new metastatic lesions to test for MDM2/MDM4 amplification.

Saada-Bouzid and others, in their study questioned the role of prior radiation therapy in hyperprogression, as most of the cases studied in their paper had at least a locoregional recurrence in an irradiated field [18]. Ogata et al. in their reported case of GEJ tumor hyperprogression also made note of the recent radiation therapy before ICI initiation in the patient reported, and wondered if the radiotherapy could have induced tumor antigen production which altered the immune environment and facilitated hyperprogression [1]. This is also not in line with our case report in which there is a significant time interval between radiotherapy and initiation of ICI.

In 2019 Lo Russo and others studied 187 non-small cell lung cancer patients treated with ICIs and of these 39 patients were identified as hyperprogressors [19]. Interestingly all of the hyperprogression patients in this study had pretreatment tissue samples showing tumor infiltration by a specific immunophenotype of epithelioid macrophages which co-expressed CD163, CD33, and PD L1. Further, in animal models, they demonstrated hyperprogression in mice treated with anti PD1 but not with anti PD1 F(ab)2 fragments which lacked the Fc component, thereby implicating the Fc component of anti PD1 agent as the crucial factor for hyperprogression. This study suggested Fc receptor engagement of ICI possibly on clustered CD163+CD33+PDL1+ epithelioid macrophages as a likely triggering mechanism for hyperprogression.

The most recently proposed hypothesis is put forth by Kamada and colleagues, who examined hyperprogression in gastric cancer patients, and on analyzing tumor samples before and after therapy noted markedly increased Ki67+ effector T regulatory cells (FoxP3CD45RA-CD4+) in majority of hyperprogressive patients after ICI therapy [20]. In vitro studies also showed that either genetic ablation or antibody mediated blockade of PD1 in T regulatory cells increased the proliferation of these cells with a noted suppression of antitumor immune response. Given the known immunosuppressive role of T regulatory cells, the PD1 blockade on these cells causing enhanced immunosuppression could also explain hyperprogression.

## Conclusions

Our patient’s dramatically improved status of immunotherapy, after radiologic evidence of hyperprogression, adds to the growing pool of literature on this critical complication of ICIs. We encourage further studies to investigate the mechanism underlying this phenomenon. It is also hoped that future research can identify conclusively if there are definite risk factors in patient populations that makes them particularly prone to hyperprogression. This should help improve the selection of patients best suited to checkpoint inhibitor therapy so as to avoid this alarming repercussion.
